# Chronic Bright’s Disease, with Renal Calculi, in a Bitch

**Published:** 1894-01

**Authors:** Andrew Darling


### CHRONIC BRIGHT'S DISEASE, WITH RENAL CALCULI, IN A BITCH.

By Andrew Darling, M.D., D.V.S.

The following notes of a case of chronic Bright's disease may prove of interest. This disease is evidently very rare in the canine species; at least, I have failed to find it mentioned in any available work on the diseases of the dog. An interesting feature of the case is the long existence of a gastric catarrh, shown by the vomiting and finally haematemesis, in this respect resembling the symptoms occasionally seen in the disease in the human family.

The patient was a water spaniel bitch, age six years, the property of a livery man. The history given was as follows: On the evening of March 28th, 1893, she was noticed to vomit food, for which nothing was done. The next morning she was found vomiting blood and evidently suffering a great deal; this continued at irregular intervals, and some time later she was found sitting in a puddle of water. A neighbor's dog having recently died with symptoms of poisoning, the same suspicion was entertained in this case. When seen at 11 A. M. she was very weak and barely able to drag herself along. This became more pronounced, until late in the afternoon she was unable to get up, but consciousness was retained as evinced by wagging her tail when spoken to. About 5 P. M. she became comatose, and died, after a few struggles, within an hour.

After the post-mortem the following additional facts were gathered from the stable help: She had always been a ravenous eater, getting three meals a day, principally of meat; appetite began to fail a few days before sickness. Until about three months ago had been in fat condition, but at that time began gradually to lose flesh; this was specially noticeable three weeks ago, after her return from the country, where she had been for a week to be "lined." Occasional vomiting of food had been noticed for many months; other important points were incontinence of urine and frequent micturition.

A rough post-mortem examination was made, the important points of which were as follows: The stomach and all the intestines, except lower part of colon and rectum, were deeply congested, and when opened found to contain a dark bloody mucus, but no ulceration. The left ventricle of heart was greatly hypertrophied; but there was no valvular disease. On removing a large amount of adipose tissue from around the kidneys, the great difference in size and their nodulated appearance was strikingly apparent. The left kidney weighed 1110 grains, and the right 661 grains. The capsule was firmly adherent in many places. On section they gave the peculiar grating sound due to the increased amount of fibrous tissue. A yellowish-colored calculus was found in the pelvis of each kidney, that from the left weighing 44 grains, and the right 21 grains. To the naked eye, chronic interstitial nephritis predominates, but changes indicating parenchymatous nephritis and fatty degeneration are also present. The specimens have been forwarded to Prof. Wesley Mills of McGill College, Montreal, for further examination.

